# Muscle p70S6K phosphorylation in response to soy and dairy rich meals in middle aged men with metabolic syndrome: a randomised crossover trial

**DOI:** 10.1186/1743-7075-11-46

**Published:** 2014-09-30

**Authors:** Petra Gran, Amy E Larsen, Maxine Bonham, Aimee L Dordevic, Thusitha Rupasinghe, Claudio Silva, Amsha Nahid, Dedreia Tull, Andrew J Sinclair, Cameron J Mitchell, David Cameron-Smith

**Affiliations:** Molecular Nutrition Unit, School of Exercise and Nutrition Sciences, Deakin University, Burwood, Victoria Australia; Metabolomics Australia, University of Melbourne, Parkville, Victoria Australia; School of Medicine, Deakin University, Waurn Ponds, Victoria Australia; The Liggins Institute, Faculty of Medical and Science Health, University of Auckland, 85 Park Road, Grafton, Private Bag 92019, Auckland, 1023 New Zealand

## Abstract

**Background:**

The mammalian target of rapamycin (mTOR) pathway is the primary regulator of muscle protein synthesis. Metabolic syndrome (MetS) is characterized by central obesity and insulin resistance; little is known about how MetS affects the sensitivity of the mTOR pathway to feeding.

**Methods:**

The responsiveness of mTOR pathway targets such as p706Sk to a high protein meal containing either dairy or soy foods was investigated in healthy insulin sensitive middle-aged men and those presenting with metabolic syndrome (MetS). Twenty male subjects (10 healthy controls, 10 MetS) participated in a single-blinded randomized cross-over study. In a random sequence, subjects ingested energy-matched breakfasts composed predominately of either dairy-protein or soy-protein foods. Skeletal muscle biopsies were collected in the fasted state and at 2 and 4 h post-meal ingestion for the analysis of mTOR- and insulin-signalling kinase activation.

**Results:**

Phosphorylated Akt and Insulin receptor substrate 1 (IRS1) increased during the postabsorptive period with no difference between groups. mTOR (Ser448) and ribosomal protein S6 phosphorylation increased 2 h following dairy meal consumption only. p70S6K (Thr389) phosphorylation was increased after feeding only in the control subjects and not in the MetS group.

**Conclusions:**

These data demonstrate that the consumption of a dairy-protein rich but not a soy-protein rich breakfast activates the phosphorylation of mTOR and ribosomal protein S6, required for protein synthesis in human skeletal muscle. Unlike healthy controls, subjects with MetS did not increase muscle p70S6K(Thr389) phosphorylation in response to a mixed meal.

**Trial registration:**

This trial was registered with the Australian New Zealand Clinical Trials Registry (ANZCTR) as ACTRN12610000562077.

## Introduction

Skeletal muscle is the most abundant tissue in the human body, aside from its primary role in locomotion it is also the primary site of oxidative metabolism, insulin-stimulated glucose uptake [1] and amino acid uptake [2]. Muscle size is regulated by the balance between muscle protein synthesis (MPS) and muscle protein breakdown (MPB) [3]. In the fasted state MPS is suppressed and MPB is elevated such that the muscle is in a net catabolic state [4]. In young healthy individuals the ingestion of a protein containing meal results in an elevation in MPS and a suppression of MPB resulting in a net anabolic state [4]. In healthy young individuals postabsorptive catabolism and postprandial anabolism are equal in the long term and muscle size is maintained [3]. MPS appears to be much more tightly regulated than MPB [5] and is primarily regulated though the mammalian target of the rapamycin (mTOR) pathway which is sensitive to the effects of growth factors such as insulin, nutrients and contraction [6]. The branched chain amino acid (BCAA) leucine has been shown to be the primary nutrient regulator of the mTOR pathway [7].

The consumption of intact protein or an amino acid mixture containing leucine results in a robust phosphorylation of p70S6 kinase (p70S6K), a downstream target of the mTOR pathway [8–10]. p70S6K phosphorylation leads to increases in the initiation of protein translation and ultimately MPS. Short term physical inactivity appears to lead to a decreased MPS response to protein feeding; however, changes in the response of the mTOR pathway are less clear [11–13]. In many but not all studies, old age is associated with an ‘anabolic resistance’ to protein feeding where MPS is not elevated in response to feeding in the elderly. This anabolic resistance appears to be mediated through decreased sensitivity of the mTOR pathway to feeding [9, 14]. It is unclear if ageing *per se* is the cause of impaired anabolic signalling or if a decline in physical activity and a different metabolic phenotype are the underlying causes of the observed anabolic signalling deficits.

Many studies have examined the effects of free amino acid or intact protein ingestion on MPS and mTOR signaling [9, 15].

These studies have shown that proteins high in the BCAA leucine result in greater mTOR activation compared with proteins containing less leucine [8, 15–17]. Dairy and soy food are sources of dietary protein; because of its greater leucine content, milk protein appears to results in a greater MPS response than soy protein, at least after resistance exercise [18]. There have been a few recent studies which have examined MPS and mTOR pathway response to the ingestion of mixed macronutrient beverages; however, there is very little research on the ingestion of true mixed meal on activation of the mTOR pathway [15, 19]. There is evidence that the addition of fat, carbohydrate and fiber to protein may slow the appearance of amino acids in the blood and thus blunt the anabolic signalling response [20–22]. There is also evidence that consuming solid food rather than a liquid supplement results in a slower rate of appearance of amino acids in the blood [22].

Metabolic syndrome (MetS) is a characterized by a cluster of conditions which include central obesity, dyslipidemia, high blood pressure and insulin resistance [23]. There has been little research on MetS and protein metabolism; however, older type 2 diabetics display lower muscle strength, mass, and quality compared to age matched controls [24]. Type 2 diabetics also display an insulin resistance of protein metabolism at the whole body level [25]. However, they are able to normally activate some members of the mTOR pathway in muscle during a hyperinsulinaemic hyperaminoacidaemic clamp [26]. Little is known about the muscle anabolic response of those with MetS under more physiological conditions such as mixed meal consumption.

Therefore the objectives of the current study were three fold. Firstly, to determine whether ingestion of a single breakfast meal results in the activation of the mTOR signalling pathway in middle aged men. Secondly, to examine whether meals differing in amino acid composition, yet matched for total energy and macronutrient composition, result in altered mTOR signalling. Lastly, to investigate if middle aged men with MetS display a resistance of anabolic signalling to mixed meal ingestion compared with healthy controls.

## Methods

### Subjects

A total of 20 men (*n* = 10 healthy controls, *n* = 10 MetS) aged between 40–60 years were recruited from newspaper, poster, and flyer advertisements to participate. Subjects were classified as having MetS based on the International Diabetes Federation criteria [27]. They had to present with abdominal obesity (waist circumference ≥ 94 cm) and two of the following factors: raised serum triglycerides (≥1.7 mmol/l), reduced serum HDL cholesterol (<1.03 mmol/l), impaired fasting glycaemia (fasting plasma glucose ≥5.6 mmol/l) or raised blood pressure (systolic blood pressure ≥130 mmHg or diastolic blood pressure ≥85 mmHg). A cohort of age and height matched healthy controls, without MetS were also included. Subjects were excluded if they showed evidence of acute or chronic inflammatory disease, infectious diseases, cancer, and/or known alcohol consumption (>20 g per day). Subjects with fasting glucose concentrations indicative of T2DM were also excluded along with people on diabetic medications. All experimental procedures were performed in accordance with the Helsinki declaration of 1975 as revised in 1983 and were formally approved by the Deakin University Human Research Ethics Committee (EC-120, 2008). Informed written consent was obtained from each subject before participation in the study and after the nature, purpose, and risks of the study were explained and subjects informed of their right to withdraw from the trial at any stage of the investigation.

### Experimental design

A controlled crossover single meal study was conducted to examine the postprandial effects to either a dairy or soy meal, with at least four weeks wash out between the study days. Subjects were instructed to abstain from alcohol, caffeine and tobacco on the day proceeding the trial day. On the morning of the trial, subjects presented to the Deakin University clinical laboratory in a fasted state. Upon arrival, they had their height, weight and blood pressure measured. A cannula was inserted in the antecubital vein and a fasting blood sample was collected. Blood samples were collected 30 min, 60 min, 180 min and 240 min following the test meal for plasma amino acid analysis. Following 30 min of supine resting, a muscle sample was collected from the *vastus lateralis* under local anesthesia (Xylocaine 1%) by percutaneous needle biopsy technique [28] modified to include suction [29]. Muscle tissue from each biopsy was immediately frozen and stored in liquid nitrogen for later analysis. Following this, subjects were required to consume a high fat dairy or high fat soy meal within approximately 15 min. The details of the study design and meal have been previously reported [30]. Further muscle samples were collected at 2 h and 4 h post meal ingestion with serial biopsy samples collected at least 2 cm from previous biopsy sites.

### Test meals

Subjects were randomly assigned to consume either a breakfast meal comprised of dairy-derived protein or void of dairy-derived proteins (replaced with the same level of soy based protein). The interval between the two test meals was at least four weeks. To prevent possible differences between subjects at baseline from their previous meal the night prior to the study day, subjects were provided with a controlled meal for dinner. The meal consisted of a pre-packaged lasagna and fruit yogurt providing a total of 2462 kJ as 20% fat, 18% protein and 62% carbohydrates. Subjects were asked to eat only the provided food and nothing else. The test breakfast meals consisted of cheese, butter, and full cream milk with white bread toast (dairy breakfast) and the second meal contained soy cheese analogue, soy beverage, a soy spread, and white bread toast and contained the same amount of protein (31 g) with similar carbohydrate content (Table 1). As the test meals contained different sources of protein, the amino acid composition also differed (refer to Table 2). Subjects were asked to consume the entire breakfast meal within 15 min.

**Table 1:** **Energy content and macronutrient composition of the high fat meals**

Meal	Composition	Amount	Total energy (kJ)	Protein (g)	Fat (g)	Carbohydrate (g)	Sodium (mg)
**Soy**	Margarine	20 g	3276	31	54	48	1297
	Full fat soy milk	300 mL					
	Soy cheese	100 g					
	Bread (white)	50 g					
**Dairy**	Butter	23 g	3120	31	54	37	951
	Full cream milk	300 mL					
	Cheese	70 g					
	Bread (white)	50 g

**Table 2:** **Amino acid composition of the test meals**

Amino Acid	Soy		Dairy
		mg/g (%)	
Histidine	2.1		2.3
Serine	6.7		6.8
Arginine	5.2		2.8
Glycine	7.2		3.8
Aspartic acid	9.5		6.4
Glutamic acid	21.5		21.6
Threonine	4.0		4.1
Alanine	5.9		4.5
Proline	8.1		11.8
Lysine	4.8		6.2
Tyrosine	2.1		3.0
Methionine	1.1		2.2
**Valine**	**5.3**		**6.6**
**Isoleucine**	**4.5**		**4.7**
**Leucine**	**7.8**		**9.1**
Phenylalanine	4.3		4.0
**Total BCAA**	**17.6**		**20.4**

### Quantitative amino acid analyses of test meals

Test meals were homogenized and underwent liquid hydrolysis in 6 M HCl at 110°C for 24 h. Following hydrolysis, amino acid derivation was conducted using AccQ-Tag reagents as per manufacturer’s instructions. Liquid chromatographic analysis was performed on a Waters Acquity UPLC system, equipped with a binary solvent manager, an autosampler, a column heater, a PDA detector, and interfaced to a tandem quadrupole detector at the Australian Proteome Analysis Facility (APAF; Macquarie University, New South Wales, Australia) [31].

### Anthropometric measurements

Weight, height and waist circumference were measured at baseline and upon arrival to the clinical laboratory following an overnight fast on test days. Blood pressure was measured on three occasions using a mercury sphygmomanometer.

### Biochemical measurements

Venous blood samples were drawn at fasting, 30 min, 60 min, 180 min, and 240 min after consumption of the meal using EDTA tubes. EDTA blood samples were centrifuged at 3000 *rpm* for 15 min and 300 μL of separated plasma removed for subsequent amino acid analysis. Blood samples were also collected in serum tubes, centrifuged at 3000 *rpm* for 15 min, stood for 30 min at room temperature, and were then supplied to Cabrini Pathology (Cabrini Health, Victoria, Australia) for assessment of insulin and glucose. To estimate insulin resistance, HOMA index was calculated by the formula: HOMA = (fasting plasma insulin in μU/mL × fasting plasma glucose in mM)/22.5 [32, 33].

### Plasma amino acids

Quantification of plasma amino acids was carried out by reverse-phase high-performance liquid chromatography (HPLC) with pre-column derivitisation of plasma samples with purified 6-aminoquinolyl-*N*-hydroxysuccinimidyl carbamate (AQC; School of Botany, University of Melbourne, Victoria, Australia) followed by reverse-phase HPLC [31]. Briefly, 50 μL of plasma was incubated with 100 μL of chilled acetonitrile (ACN) to precipitate plasma proteins. Samples were briefly vortexed and centrifuged for 10 min at 0°C before 10 μL of the resulting supernatant was aliquoted into an HPLC vial with glass insert for derivitisation. Seventy microlitres of borate buffer was then added to each sample and subsequently mixed prior to the addition of 20 μL of 3 mg/mL (w/v) AQC. Derivitisation reaction occurred with heating at 55°C for 10 min with gentle agitation at 750 *rpm*. Separation of samples was achieved using an Agilent 1200 Series LC system with a Binary SL pump (Agilent Technologies, California, USA). AQC derivatives were subsequently detected using an Agilent 6460 Triple Quad LC/MS with dynamic MRM detection. All samples were analysed using Agilent Masshunter Quantitative Analysis software, version B.03.02, 2008 (Agilent Technologies). Plasma amino acid concentrations were determined by measuring the absolute area under the curve relative to a standard curve generated using preparations of an internal standard (2-aminobutyric acid; data not shown). All samples from an individual subject were analysed within the same assay in a randomised order.

### Immunoblotting

Approximately 30 mg of muscle was homogenized, in lysis buffer (pH 7.0) containing 20 mM Tris–HCl, 5 mM EDTA, 10 mM Na-pyrophosphate, 100 mM NaF, 2 mM Na3VO4, 1% Igepal, 10 g/mL Aprotinin, 10 g/mL Leupeptin, 3 mM Benzamidine and 1 mM PMSF.

The homogenate was rotated for 1 h at 4°C and subsequently centrifuged at 14,000 rpm for 15 min with the resulting supernatant collected into a fresh tube. Protein concentrations were determined using a BCA Protein Assay (Pierce, Thermo Scientific, New South Wales, Australia) with bovine serum albumin (BSA) as a standard. Muscle homogenate was then denatured in loading buffer containing dithiothreitol (DTT) and separated by sodium dodecyl sulphate-polyacrylamide gel electrophoresis (SDS-PAGE). Proteins were then transferred onto nitrocellulose membrane and blocked at room temperature using 5% BSA (Sigma-Aldrich) in tris buffered saline with 0.1% (v/v) Tween-20 (TBST; Sigma-Aldrich). Membranes were incubated overnight with antibodies in 5% BSA at 4°C. Expression of signalling kinases was determined using primary antibodies (1:1,000) specific for IRS1^Tyr612^, Akt^Ser473^, mTOR^Ser2448^, p70S6K^Thr389^, and ribosomal S6^Ser240/244^ (Cell Signaling). Membranes were subsequently washed and then incubated with anti-rabbit HRP-conjugated secondary antibody (1:1000; Calbiochem) for 1 h at room temperature. Following this, membranes were washed repeatedly as before, and proteins visualized using enhanced chemiluminescence (Perkin-Elmer, Queensland, Australia) on a Kodak 4000MM Image Station (Kodak, New York, USA) using a CCD camera. Membranes were stripped using Restore Western Blot Stripping buffer™ (Quantum Scientific, Victoria, Australia) and then subsequently reprobed for total mTOR, total p70S6K, and total eIF4G proteins to verify the relative amount of analyzed proteins. Band density was quantified using Kodak imaging software version 4.5.0 (Kodak); each phosphorylated protein was normalized to its respective total protein.

### Statistical analysis

Statistical analysis was performed using SPSS version 20 for Windows (SPSS Inc.). Data are reported as means ± standard error of the mean (SEM). Paired Students *t*-test with Bonferroni adjustment was used to determine significance of between group differences at baseline. A two-way analysis of variance (ANOVA) with group (control, MetS) and meal (dairy, soy) as factors was used to test for BCAA area under the curve (AUC) difference. Differences in all other variables were tested using a three-way ANOVA with group (control, MetS) and meal (dairy, soy) as between subject factors and time as a within subject factor. The Holm-Sidak post hoc method was used to compare pair wise differences when interactions were present. A probability level of <0.05 was adopted throughout to determine statistical significance.

## Results

### Amino acid composition of test meals

There were slight differences in the relative abundances of each amino acid between the soy and dairy meal (Table 2). Leucine concentration was 1.2-fold higher in the dairy meal compared to the soy meal. Similarly, the total BCAA content of the dairy meal was 1.2-fold greater than the soy meal yet matched for total protein.

### Plasma concentrations of insulin and glucose

**Figure 1:**
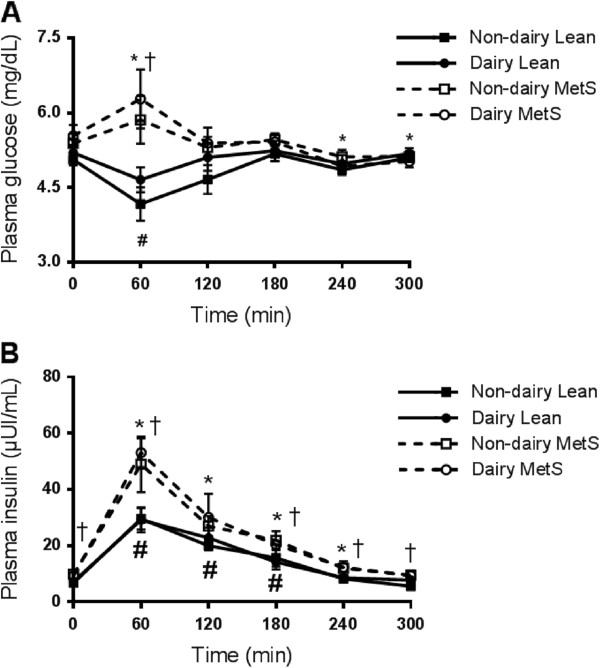
**Postprandial time course of plasma insulin and glucose.** Insulin (μU/mL) **(A)** and glucose (mg/dL) **(B)** concentrations were assessed in plasma collected from healthy control and metabolic syndrome subjects following consumption of a high fat soy or dairy breakfast. Healthy control subjects are represented with solid lines and closed squares (■) for the dairy meal and closed circles (●) for the soy meal. Metabolic syndrome (MetS) subjects are represented with dashed lines and open squares (□) for the dairy meal and open circles (○) for the soy meal. The graphs represent mean values ± SEM; *n* = 10 per group. * = Significantly different than baseline within the Metabolic syndrome subjects irrespective of meal *P* < 0.05. # = significantly different from baseline within healthy control subjects irrespective of meal *P* < 0.05. † = Significant difference between subjects with metabolic syndrome and healthy control irrespective of meal *P* < 0.05.

Baseline plasma insulin concentrations were not different between the alternate meal days within each group. Glucose concentration (Figure 1A) did not differ between groups at baseline; however, there was a group X time interaction (P < 0.001) such that plasma glucose was elevated above baseline in the MetS group at 60 min after the meal and was below baseline at the same time point in the healthy control group irrespective of meal. Fasting blood insulin concentrations were significantly greater in the MetS subjects compared with their healthy control counterparts (9.6 ± 0.1 and 6.1 ± 0.5 respectively; *P* < 0.01; Table 3). There was a group main effect (P = 0.005) and group X time (P = 0.027) interaction in plasma insulin concentration such that insulin concentration was greater in the MetS subjects at every time point with the exception of 120 min; the difference was largest at 60 min after consumption of the meal (Figure 1B). Furthermore, subjects presenting with MetS appeared to be less insulin sensitive (i.e. more insulin resistant) than the healthy control subjects. The homeostasis model of assessment (HOMA) index was 1.8-fold higher in MetS subjects compared with the healthy controls (*P* < 0.01; Table 3). There were no meal effects (glucose: P = 0.49, insulin: P = 0.066) or meal X time interactions (glucose: P = 0.49, insulin: P = 0.51).

**Table 3:** **Baseline characteristics and physiological measures** ^**1**^

	Healthy *n* = 10	Metabolic syndrome *n* = 10	P value
Age (years)	51.2 ± 1.4	53.1 ± 1.5	0.359
Weight (kg)	83.1 ± 1.9	102.2 ± 5.5	0.004
BMI (kg/m^2^)	26.6 ± 0.8	33.5 ± 1.9	0.003
Waist circumference (cm)	93.6 ± 2.3	111.9 ± 3.8	0.000
HOMA index	1.4 ± 0.1	2.5 ± 0.3	0.007
Fasting insulin (μUI/mL)	6.1 ± 0.5	9.6 ± 1.0	0.005
Fasting glucose (mg/dL)	5.1 ± 0.1	5.9 ± 0.4	0.101

^*1*^All results presented as means ± SEM.

### Plasma concentrations of amino acids

**Figure 2:**
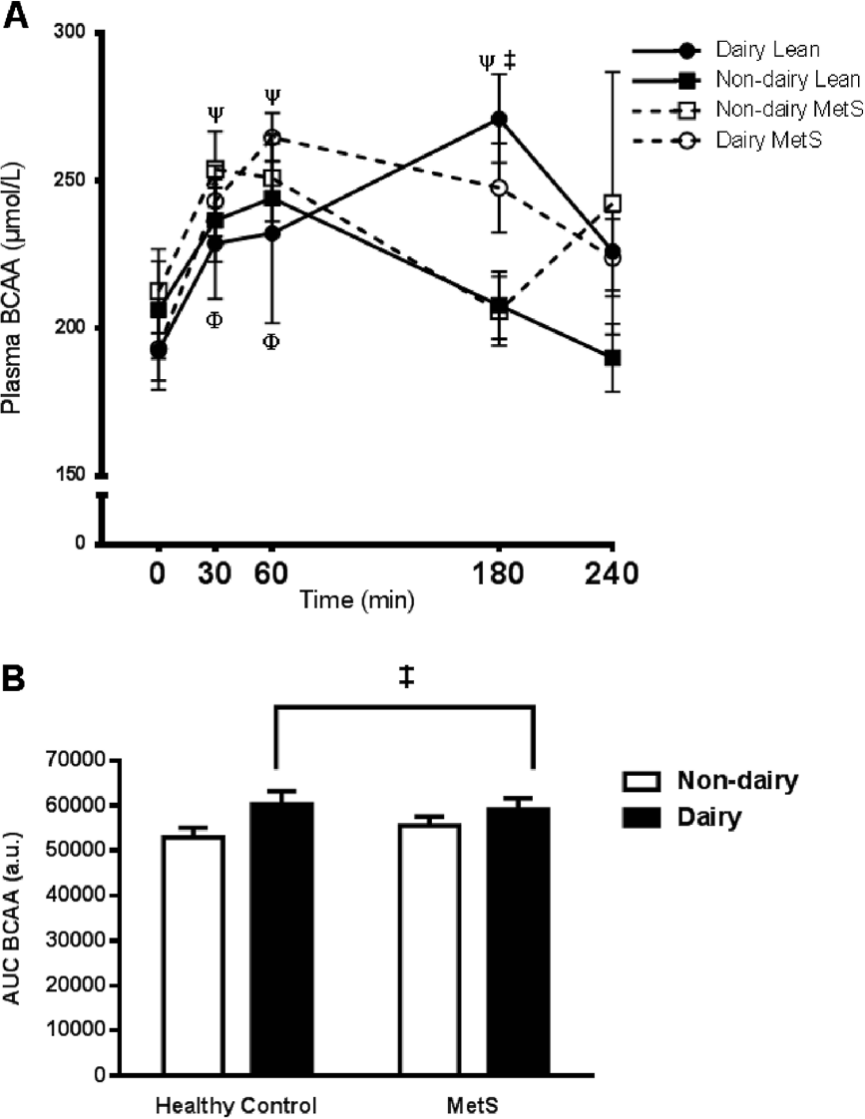
**Plasma BCAA response to mixed-meal ingestion.** Plasma concentration of the three branched chain amino acids (BCAA) leucine, isoleucine and valine were measured in healthy control and metabolic syndrome subjects following consumption of a high fat soy or dairy breakfast. **A)** Time course of BCAA concentrations; healthy control subjects are represented with solid lines and closed squares (■) for the dairy meal and closed circles (●) for the soy meal. Metabolic syndrome (MetS) subjects are represented with dashed lines and open squares (□) for the dairy meal and open circles (○) for the soy meal. **B)** Area under the curve (AUC); open bars represent soy and closed bars represents dairy meals. The graphs represent mean values ± SEM; *n* = 10 per group. Ψ = significantly different from baseline within the soy condition irrespective of group *P* < 0.05. Φ = significantly different from baseline within the dairy condition irrespective of group *P* < 0.05. ‡ = Significant difference between the dairy and soy conditions irrespective of group *P* < 0.05.

In order to elucidate differences in the plasma amino acid profile after consumption of a single mixed meal, plasma amino acid concentrations were measured at 0, 30, 60, 180, and 240 min in both healthy control and MetS subjects. Table 4 (control) and Table 5 (MetS) show the plasma concentrations over time of all the individual amino acids which could be resolved with the HPLC. Total plasma amino acid concentration did not differ between groups but did differ between meals (meal X time, P = 0.047); greater total amino acid concentration was observed 180 min after the dairy meal compared with the soy meal. Figure 2A shows the plasma BCAA (leucine, isoleucine, valine) response to the dairy and soy meals in control and MetS subjects. BCAAs followed the same pattern as total amino acids such that there was a greater BCAA concentration following the dairy meal at 180 min post ingestion compared to the soy meal irrespective of group (meal X time, P = 0.047). A greater AUC for BCAAs was observed following the dairy meal compared with the soy meal irrespective of subject group (P < 0.001; Figure 2B).

**Table 4:** **Time course changes in plasma concentrations of selected amino acids in healthy controls** ^**1**^

	Soy (*n* = 10)						Dairy (*n* = 10)			
	Time (min)									
	0	30	60	180	240	0	30	60	180	240
	*μ*mol/L									
**Arginine**	11.0 ± 1.0	14.6 ± 2.3	13.0 ± 1.1	10.7 ± 0.8	10.3 ± 1.4	9.6 ± 0.9	9.3 ± 0.5	10.7 ± 0.9	11.3 ± 1.5	8.5 ± 0.8*
**Asparagine**	7.9 ± 1.0	9.8 ± 0.6	9.9 ± 0.5	7.5 ± 0.4	6.7 ± 0.6	7.0 ± 0.5	7.1 ± 0.5	8.4 ± 1.0	8.5 ± 0.8	6.5 ± 0.5
**Serine**	15.8 ± 2.0	17.1 ± 1.5	16.9 ± 1.1	14.1 ± 0.8	11.0 ± 0.5	14.1 ± 1.5	13.4 ± 1.2	16.4 ± 1.7	16.4 ± 1.4	13.8 ± 0.9
**Glutamine**	95.3 ± 2.4	101.7 ± 3.9	108.2 ± 2.2**	105.0 ± 6.4	96.9 ± 4.4	104.9 ± 6.2	96.7 ± 3.7	111.0 ± 6.8	121.8 ± 9.1	105.0 ± 5.4
**Taurine**	10.2 ± 0.8	9.9 ± 1.0	10.2 ± 1.2	9.3 ± 1.3	10.5 ± 1.2	10.8 ± 1.1	7.6 ± 0.4**	8.5 ± 1.0	9.8 ± 0.9	9.6 ± 0.8
**Glycine**	43.4 ± 2.1	48.0 ± 3.0	47.5 ± 2.0	46.5 ± 4.4	37.6 ± 2.9	47.5 ± 2.7	37.6 ± 2.3*	42.3 ± 3.2	43.8 ± 3.4	37.2 ± 1.6**
**Proline**	73.3 ± 4.7	86.1 ± 4.7*	93.6 ± 4.8**	82.3 ± 4.2	75.5 ± 3.9	69.6 ± 3.5	81.5 ± 3.7	91.0 ± 4.2*	112.6 ± 7.6**	92.5 ± 3.3**
**Lysine**	20.3 ± 2.5	21.7 ± 2.8	20.6 ± 1.5	18.5 ± 2.5	18.1 ± 2.5	17.4 ± 2.2	18.1 ± 1.6	22.3 ± 1.9	21.8 ± 3.2	17.5 ± 2.2
**Valine**	92.5 ± 7.0	98.0 ± 4.5*	102.6 ± 4.4*	91.4 ± 4.4	84.6 ± 5.2	89.1 ± 6.0	89.9 ± 5.0*	110.3 ± 7.5*	119.3 ± 6.3**	103.4 ± 5.5*
**Isoleucine**	35.0 ± 3.1	46.5 ± 3.3	47.3 ± 2.9*	39.4 ± 2.5	36.9 ± 2.5	31.3 ± 2.5	39.0 ± 2.6**	46.7 ± 3.5*	48.1 ± 3.0*	36.6 ± 1.8
**Leucine**	80.3 ± 6.2	92.2 ± 5.0	91.9 ± 4.5	74.8 ± 3.7	68.4 ± 4.2	72.0 ± 4.9	84.6 ± 4.8**	101.0 ± 7.3	103.7 ± 6.0*	83.9 ± 5.3
**∑BCAA**	206.2 ± 17.3	236.7 ± 16.3	241.7 ± 16.9**	205.5 ± 15.3	190.0 ± 14.0	192.4 ± 17.2	228.8 ± 17.1	257.9 ± 19.8*	265.4 ± 20.4*	226.1 ± 19.1*
**∑Total AA** ^**2**^	545.5 ± 52.9	577.0 ± 28.9	597.3 ± 20.6	526.5 ± 25.8	486.6 ± 28.8	505.8 ± 29.0	527.5 ± 28.9	597.5 ± 37.1*	658.2 ± 38.7**	540.5 ± 24.0*

^*1*^Means which are statistically different from baseline (0 min) for each meal are indicated by: * *P* < 0.05, ** *P* < 0.01.

^2^Total AA: sum of measurable amino acids.

**Table 5:** **Time course changes in plasma concentrations of selected amino acids in subjects with metabolic syndrome** ^**1**^

	Soy (*n* = 10)					Dairy (*n* = 10)				
	Time (min)									
	0	30	60	180	240	0	30	60	180	240
	*μ*mol/L									
**Arginine**	8.7 ± 0.7	11.3 ± 0.8**	11.9 ± 1.0**	9.3 ± 0.9	8.5 ± 0.8	8.6 ± 0.4	9.6 ± 0.5	10.5 ± 0.7*	8.9 ± 0.6	7.7 ± 0.4
**Asparagine**	6.3 ± 0.8	8.7 ± 0.8**	8.4 ± 0.8**	6.4 ± 0.4	6.3 ± 0.5	6.3 ± 0.4	7.2 ± 0.5	8.0 ± 0.4**	6.6 ± 0.4	5.9 ± 0.2
**Serine**	12.9 ± 1.7	13.3 ± 0.8	15.2 ± 1.5	12.3 ± 1.6	11.5 ± 1.1	13.3 ± 1.4	15.4 ± 1.4	15.8 ± 1.4**	13.8 ± 1.4	12.7 ± 1.4
**Glutamine**	99.5 ± 7.0	90.9 ± 7.4*	95.6 ± 6.7	89.6 ± 4.1*	94.3 ± 4.8	99.7 ± 6.4	95.7 ± 6.5	103.1 ± 4.7	102.7 ± 6.5	103.4 ± 7.0
**Taurine**	8.6 ± 0.8	7.7 ± 0.5	8.0 ± 0.6	8.5 ± 0.6	8.6 ± 0.5	8.8 ± 0.5	7.4 ± 0.3*	8.3 ± 0.3	8.3 ± 0.5	8.2 ± 0.4
**Glycine**	36.7 ± 3.6	36.6 ± 3.4	37.4 ± 3.5	33.4 ± 2.5*	37.1 ± 4.8*	40.2 ± 3.8	35.8 ± 2.2	38.5 ± 2.5	34.5 ± 2.5**	32.9 ± 2.4*
**Proline**	65.8 ± 3.8	79.6 ± 5.5**	86.7 ± 7.8**	74.6 ± 4.6**	74.4 ± 6.3	68.5 ± 3.8	83.2 ± 5.0*	101.6 ± 5.8**	99.4 ± 5.9**	88.1 ± 5.9**
**Lysine**	16.6 ± 1.7	19.6 ± 2.5	19.4 ± 2.4	15.6 ± 2.3	16.8 ± 2.0	15.1 ± 1.1	20.5 ± 2.4*	18.6 ± 1.6**	19.8 ± 2.9	18.0 ± 2.1
**Valine**	99.0 ± 7.2	108.0 ± 6.5**	109.4 ± 7.6*	95.3 ± 6.3	93.2 ± 4.4	88.9 ± 4.7	101.3 ± 4.9*	112.2 ± 4.0**	111.1 ± 6.6**	103.4 ± 5.1**
**Isoleucine**	35.4 ± 2.6	47.6 ± 2.6**	47.8 ± 3.1**	38.4 ± 2.4	36.5 ± 1.7	31.7 ± 2.1	44.4 ± 2.8**	47.7 ± 2.2**	43.0 ± 3.2**	38.2 ± 3.1*
**Leucine**	78.2 ± 4.9	98.3 ± 4.4**	93.7 ± 4.8**	72.2 ± 3.6*	68.8 ± 2.6*	70.4 ± 4.9	100.6 ± 4.3**	103.5 ± 2.2**	90.4 ± 5.8**	83.8 ± 4.8*
**∑BCAA**	212.6 ± 18.7	253.9 ± 18.7*	251.0 ± 18.5*	205.9 ± 16.5	242.4 ± 21.1	196.2 ± 17.8	243.1 ± 18.4	264.8 ± 20.4*	247.6 ± 20.4*	224.0 ± 19.2*
**∑Total AA** ^**2**^	538.0 ± 62.8	585.7 ± 56.9**	605.4 ± 69.1**	515.6 ± 51.1	527.2 ± 125.8	489.0 ± 24.3	546.5 ± 24.7	602.4 ± 21.5**	569.7 ± 31.1**	527.2 ± 32.5

^*1*^Means which are statistically different from baseline (0 min) for each meal are indicated by; * *P* < 0.05, ** *P* < 0.01.

^2^Total AA: sum of measurable amino acids.

### Protein phosphorylation

**Figure 3:**
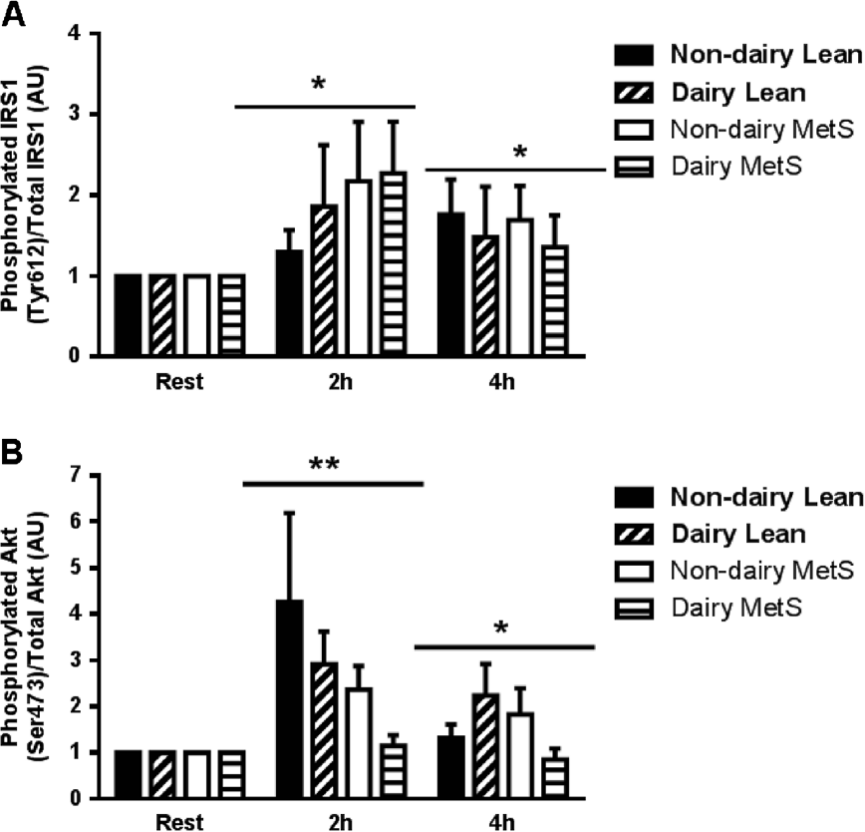
**Activation of IRS1and Akt during the postprandial period in human skeletal muscle.** Phosphorylation is normalized to the total content of each protein and expressed in arbitrary units. IRS (Tyr612) **(A)** and Akt (Ser473) **(B)** phosphorylation are shown of healthy control and metabolic syndrome (MetS) subjects following consumption of a high fat soy or dairy breakfast. The graphs represent mean values ± SEM; *n* = 10 per group. ** = Significant main effect for time, different than baseline and 4 h irrespective of group or meal *P* < 0.05. * = Significant main effect for time, different than baseline irrespective of group or meal. † = Significant difference between subjects with metabolic syndrome and healthy control subjects irrespective of meal *P* < 0.05.

In order to delineate whether individuals exhibiting metabolic dysregulation have impaired insulin signalling, the phosphorylation response of IRS1 to both a mixed dairy and soy meal was investigated. There were no group, or meal effects or interactions however, a main effect for time was observed (P = 0.008). Collapsed across all groups and conditions IRS1 phosphorylation on Tyr612 was increased ~2-fold at 2 h and ~1.6-fold at 4 h post meal consumption (Figure 3A). There was a trend (P = 0.07) for less phosphorylation in the healthy control group 2 h post consumption of the soy meal compared with the other conditions.

Given that mTOR-mediated anabolic signaling may also occur via insulin stimulation, activation of the upstream mediator, Akt, was examined (Figure 3B). Similarly to IRS1 there were no significant group or meal effects or interactions; however, there was a main effect for time (P = 0.006). Irrespective of group or meal, Akt protein phosphorylation on Ser473 was increased at 2 h post meal consumption and to a lesser extent at 4 h post meal consumption.

**Figure 4:**
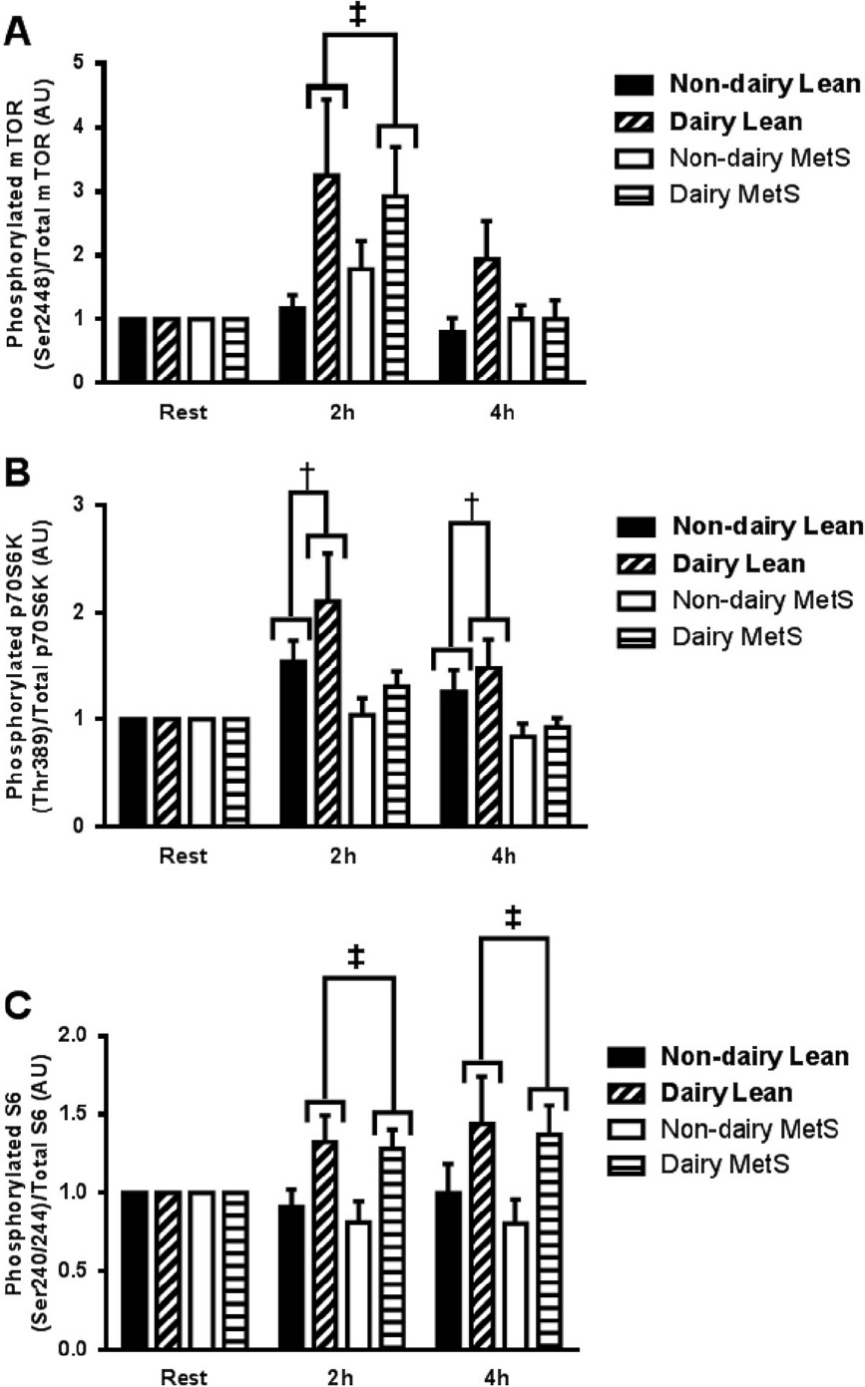
**Activation of mTOR, p70S6K and S6 during the postprandial period in human skeletal muscle.** Phosphorylation is normalised to the total content of each protein and expressed in arbitrary units. mTOR (Ser2448) **(A)**, p70S6K (Thr389) **(B)**, and **(C)** rps6 (Ser240/244) phosphorylation are shown of healthy control and metabolic syndrome (MetS) subjects following consumption of a high fat soy or dairy breakfast. The graphs represent mean values ± SEM; *n* = 10 per group. ‡ = Significant difference between the dairy and soy conditions irrespective of group *P* < 0.05. * = Significant main effect for time, different than baseline irrespective of group or meal *P* < 0.05.

In order to determine whether dairy-derived proteins can elicit greater mTOR activation in human skeletal muscle tissue, mTOR phosphorylation was evaluated. A main effect for time (P = 0.005) was observed as well as a meal effect (P = 0.05) and a trend towards a meal X time interaction (P = 0.072). Because the fold change in each group and condition was equalled at rest the group effect was indicative of an interaction even though the meal X time term in the ANOVA was only a trend. Post hoc analysis revealed in elevation in mTOR Ser2448 phosphorylation at 2 h only following the dairy meal irrespective of group (Figure 4A). No group effect (P = 0.81) or group X time interaction (P = 0.75) was observed.

To confirm that greater mTOR activation leads to increased protein translation, activation of the downstream kinase, p70S6K, was measured (Figure 4B). A time X group interaction (P = 0.046) revealed that p70S6K phosphorylation on Thr389 was increased only in the healthy controls and not in the MetS group. No meal (P = 0.28) or meal X time interactions were observed (P = 0.36). This finding indicates an anabolic resistance to mixed meal consumption in the MetS group compared to controls.

**Figure 5:**
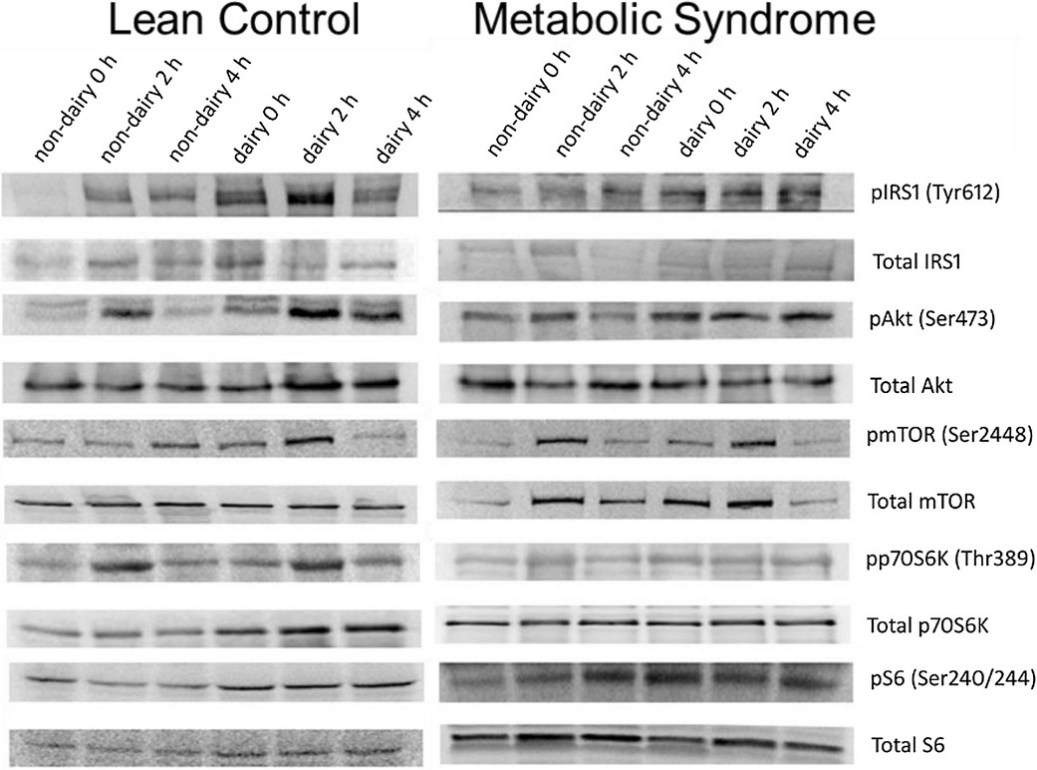
**Representative Western blots.** Example blots of protein extracted from samples of healthy control (left) and metabolic syndrome (right) subjects taken at baseline (0), 2 h, and 4 h post-ingestion of a mixed soy or dairy meal. Blots are shown for total insulin receptor (IRS1) and phosphorylation at Tyr616, total Akt and phosphorylation at Ser473, total mTOR and phosphorylation at Ser2448, total p70S6K and phosphorylation at Thr380 and total S6 and phosphorylation at Ser240/244.

Expression of phosphorylated ribosomal protein S6 kinase on Ser240/244 was elevated 2 h and 4 h following consumption of the dairy meal in both the MetS and control groups (meal X time interaction, P = 0.013). There was no elevation in ribosomal protein S6 kinase phosphorylation following the consumption of the soy meal. There was no group (P = 0.39) effect or group X time interaction (P = 0.54). Representative western blots for each of the tested proteins are shown in Figure 5.

## Discussion

The primary finding of the present study is that obese middle-aged men with metabolic syndrome but without overt type 2 diabetes show impairment in anabolic signalling following the consumption of ~30 g of protein in the context of a mixed-meal. This impairment was evidenced by a lack of p70S6K activation at 2 and 4 hours after mixed-meal consumption compared to healthy control subjects. No differences between subjects with MetS and healthy controls were observed in the activation of ribosomal protein S6 which is also a downstream target within the mTOR pathway. S6 activation and mTOR Ser2448 activation were greater following the ingestion of the dairy meal compared with the soy meal suggesting that the more sustained levels of BCAAs in the plasma following consumption of the dairy meal may result in a greater anabolic stimulus than the soy meal.

We report that when matched for total protein, fat, and similar in carbohydrate content dairy and soy based meal result in very similar blood glucose and insulin responses but that the dairy meal results in a more sustained BCAA concentration in the plasma and a larger BCAA AUC. The more sustained BCAA response is likely a result of the greater BCAA content in dairy meal and a slower release from the gut. Despite not being overtly diabetic the MetS group displayed a much larger insulin response and slightly greater glucose response to both meals compared with the control subjects. This in combination with the difference in HOMA at baseline indicates large divergence in insulin sensitivity between groups. Notwithstanding the observed differences in insulin sensitivity between MetS and controls, there were no significant differences in IRS1 or Akt phosphorylation after the consumption of either meal in either controls or MetS. Previous work has shown that type 2 diabetics display an insulin resistance to amino acid infusion [25]; however, in this study a mixed-meal does not appear to impair anabolic insulin signalling in the muscle of middle age men with MetS. It seems unlikely that insulin insensitivity is responsible for the observed downstream signalling deficit in the MetS group. However, it is possible that the addition of more subjects would have resulted in significant differences in Akt or IRS1 phosphorylation between controls and MetS. At 2 h post meal Akt phosphorylation was ~ two fold higher and IRS1 was ~ two fold lower in the controls compared to the MetS.

P70S6K is the primary readout used to assess the activity of the mTOR pathway [6, 34]; the lack of change in P70S6K phosphorylation after mixed-meal consumption in men with MetS is similar to the anabolic resistance induced by periods of inactivity [11, 12] and is observed in older adults [9, 14]. Although protein synthesis was not directly measured in this study signalling deficits have been shown to underlie ageing induced anabolic resistance [9]. In the current study we did not measure physical activity or physical fitness; however, it is likely that the MetS subjects were less physically active and fit than the controls [35]. Recent work in obese older adults has shown an insensitivity of MPS to a mixed macronutrient beverage ingestion which is transiently reversed by energy deficit and weight loss; however, the anabolic sensitivity was lost once subjects returned to their normal unsupervised diet despite maintained weight loss [36]. A possible mechanism which could underlie this anabolic resistance may be higher baseline resting phosphorylation of mTOR pathway components such as p70S6K [37]. This could be due to a constant nutrient excess in obese subjects [38, 39]. Other studies have shown higher levels of BCAAs in the obese even under fasting conditions [40]; however, we did not replicate this finding. Katta *et al*. (2009) showed diminished activation of Akt/mTOR kinases, including p70S6K, within the skeletal muscle of obese compared to lean rats following a contractile stimulus [41]. Conversely, similar rates of protein synthesis were observed in the skeletal muscle of T2DM and control subjects following administration of a high-energy clamp [42]. However, Nilsson *et al*. (2010) have demonstrated that while cytosolic and myofibrillar proteins exhibit similar rates of protein synthesis in obese and lean rats, synthesis of mitochondrial proteins is blunted in the obese group [43]. Collectively with the aforementioned studies, the current data suggests that perhaps metabolic disease results in a divergent anabolic response in skeletal muscle which is influenced by both metabolic irregularities and nutritional stimuli.

The greater S6 activation seen after the consumption of the dairy meal compared with the soy meal suggests that the greater BCAA content of dairy meal was sufficient to induce greater S6 activation. MetS did not affect S6 activation suggesting that S6 and p70S6K are regulated differently. At first it may seem surprising that mTOR phosphorylation was not increased in the healthy controls after the soy meal whereas its downstream target p70S6K was increased; however, the Ser2448 site on mTOR is actually an inhibitory site which is phosphorylated by p70S6K as a negative feedback mechanism [44]. This suggests that prior to the first post meal muscle biopsy (2 h) there may have been transient activation of the mTOR complex as well as p70S6K which was diminished by the time of sampling in the MetS group.

There have been a number of studies which have looked at the effects of different proteins on muscle anabolism however; very little research has addressed the anabolic potential of different mixed meals more closely associated with those consumed in everyday life. A fundamental characteristic of the test meals used in the present study was a large fat content (54 g). Previous research indicates that a high fat diet leads to oversaturation of the oxidative capacity of mitochondria in muscle [45]. It is unclear if the differences in mTOR and S6 activation in the present study are simply a result of different protein sources [18] and leucine content or if there is an interaction between the fat or carbohydrate and protein source.

Although this study measured anabolic signalling throughout the mTOR pathway which controls the initiation of protein translation and thus protein synthesis, acute anabolic signalling measurements do not always perfectly line up with measurements of MPS or long term phenotypic change [46, 47]. The measurement of anabolic signalling provides a ‘snapshot’ of the state of protein translation within the muscle; because measurements were made only 2 and 4 hours after meal consumption it is possible that activation prior to the first measurement or between the measurements could have been missed. Future work should directly measure MPS over time to provide an average MPS in the postprandial period.

There are many conflicting reports in the literature concerning anabolic resistance of protein synthesis and anabolic signalling deficits in ageing; even less is known about anabolic resistance or signalling deficits in middle aged men with obesity and metabolic syndrome. We report that MetS is associated with impaired downstream signalling in the mTOR pathway in response to two different high protein mixed meals. Secondarily, we report that other mTOR pathway targets are activated to a greater degree following dairy mixed consumption compared with a soy based meal matched for protein. The primary novel aspect of this study was the ingestion of a complete breakfast consisting of whole foods rather than an isolated liquid protein supplement. Future work should look to use measurements of muscle protein synthesis to directly assess differences in anabolic sensitivity in subjects with MetS using mixed meals rather than amino acid infusions and clamp methodology.

## Abbreviations

BCAA: Branched-chain amino acids
IRS1: Insulin receptor substrate 1
MetS: Metabolic syndrome
MPB: Muscle protein breakdown
MPS: Muscle protein synthesis
mTOR: Mammalian target of rapamycin
p70S6K: p70S6 kinase
S6: Ribosomal protein S6
T2DM: Type-2 diabetes mellitus.
